# A prospective cohort feasibility study of real-time beta-lactam antimicrobial therapeutic drug monitoring in critically ill patients with lower respiratory infection: The TDM-TIME study

**DOI:** 10.1177/17511437251404324

**Published:** 2025-12-24

**Authors:** Jan Hansel, Jake Lain, Emmanuel O. Erhieyovwe, Aybaniz Ismayilli, James Orr, Brian G. Keevil, Kayode Ogungbenro, Paul M. Dark, Timothy W. Felton

**Affiliations:** 1 Division of Immunology, Immunity to Infection and Respiratory Medicine, The University of Manchester, UK; 2 Critical Care, Manchester University NHS Foundation Trust, UK; 3 NIHR Centre for Precision Approaches to Combatting Antimicrobial Resistance, Manchester University NHS Foundation Trust, UK; 4 Division of Pharmacy & Optometry, The University of Manchester, UK

**Keywords:** therapeutic drug monitoring, respiratory tract infection, beta-lactams, critical illness, sepsis, microbial drug resistance, anti-bacterial agents, drug resistance

## Abstract

**Background:**

Critically ill patients with lower respiratory tract infections often fail to achieve therapeutic beta-lactam antibiotic concentrations, despite standard dosing. Therapeutic drug monitoring (TDM) may improve attaining drug exposure, but delayed turnaround times limit its clinical impact. The objective of this study was to evaluate the feasibility of delivering real-time beta-lactam TDM results within two dosing intervals.

**Methods:**

We conducted a single-centre prospective cohort feasibility study in two ICUs in Manchester, UK. Critically ill adult patients receiving piperacillin/tazobactam or meropenem for suspected or confirmed lower respiratory infection were enrolled. Blood samples were collected for analysis and drug quantification, with the primary outcome being the proportion of TDM results returned within two dosing intervals. Secondary outcomes included detailed time-to-result, clinical and microbiological outcomes. TDM results were not released to clinical teams.

**Results:**

We recruited 30 participants, of whom 20 (67%) had TDM results available within two dosing intervals. The median time from blood sampling to TDM result was 10.9 h, with a median time from antimicrobial initiation to result of 25.4 h. At 28 days, 70% of participants were alive, with a median (IQR) ICU and hospital length of stay of 7 (5–25) and 17 (10–27) days, respectively. Resistant pathogen strains were isolated in 4/21 (19%) participants.

**Discussion:**

Recruitment of critically ill participants into a time-sensitive trial of TDM is feasible. Timely feedback of beta-lactam TDM results to clinicians is achievable, however, barriers to streamlined around-the-clock implementation remain. Future clinical trials of beta-lactam TDM should factor turnaround times into study design.

**Registration:**

NCT05971979.

## Introduction

Timely interventions are crucial determinants of outcomes in critically ill patients with severe respiratory infection, including those with sepsis and septic shock.^1^ According to consensus recommendations, antimicrobial treatment should be initiated within the first few hours of suspected diagnosis, as published data suggest an association between treatment delays and increased mortality.^2,3^ Beta-lactams, the most commonly prescribed antibiotics in the intensive care unit (ICU), display highly variable pharmacokinetics (PK) in critically ill patients. Studies report that 30–50% of ICU patients fail to achieve target concentrations despite appropriate formulary-based dosing.^4^ This subtherapeutic exposure is linked to poor clinical outcomes, including treatment failure and the emergence of AMR.^5–7^ Therapeutic drug monitoring (TDM) offers a strategy to personalise antibiotic therapy by adjusting dosing based on measured drug levels.

However, randomised controlled trials (RCTs) of TDM for beta-lactam antimicrobials in ICU have not yet demonstrated a consistent mortality benefit, potentially contributed to by delays in obtaining actionable TDM results or lack of integration into bedside decision-making.^8–10^ The window of opportunity for tailored intervention in the sickest individuals is narrower, with the relative lack of reported benefit in TDM trials to date being a result of these delays. Real-time TDM, leveraging rapid assays and clinical decision support, could help address these barriers, yet feasibility data in broader ICU populations remain limited.^9,10^ Moreover, the implementation of TDM in real-world settings, including timing of blood collection, laboratory processing, and clinician notification, has not yet been extensively evaluated, with currently published studies showing delays of up to 36 h between treatment start and dose optimisation.^8^

This study aimed to investigate turnaround times for delivering actionable results from TDM of beta-lactam antimicrobials in patients admitted to the intensive care unit with severe lower respiratory infections. Furthermore, we sought to identify technology-enabled approaches to improve participant recruitment into future time-sensitive TDM trials.

## Methods

### Design and setting

This was a single-centre prospective observational cohort feasibility study conducted at two ICUs in Manchester, UK, between 12 December 2023 and 21 June 2024. The study is reported according to The Strengthening the Reporting of Observational Studies in Epidemiology (STROBE) statement.^11^ The study received a favourable opinion from the Yorkshire & The Humber - Leeds East Research Ethics Committee and the Health Research Authority (23/YH/0203). Population PK analyses were exploratory objectives of this study and will be reported elsewhere.

### Participants

We considered for inclusion: all adults aged ⩾ 18 years; admitted to ICU; treated for presumed or confirmed lower respiratory infection; receiving or about to receive the first dose of either piperacillin/tazobactam or meropenem; able to provide valid informed consent or enrol through deferred consent. We applied the following exclusion criteria: severe anaemia (haemoglobin level < 70 g/L); unlikely to survive 24 h as judged by the treating physician; and study antimicrobial course started more than 24 h ago. Severe anaemia, a common exclusion criterion in haematology and PK trials, was considered an exclusion criterion to limit the potential for harm associated with repeat blood draws.^12^ Participants were identified through an automated research notification (ARN) tool embedded in the electronic health record, designed specifically for the study. Further details on the ARN design and its assessments will be reported elsewhere.

### End points

The primary outcome was availability of liquid chromatography-tandem mass spectrometry (LC-MS/MS) results within two dose intervals of study antimicrobial administration. Dose intervals varied according to antimicrobial and indication (e.g. piperacillin/tazobactam can be administered three or four times daily, resulting in a single dose interval of 8 or 6 h, respectively). The proposed dichotomous outcome used two dosing intervals as a criterion for success, as it is possible to amend the subsequent dose within the first 24 h of an antimicrobial treatment course, if TDM is performed on the first dose. For this reason, we also assessed the proportion of participants enrolled at the point of the first dose.

Secondary outcomes were time elapsed from peripheral blood collection to LC-MS/MS result availability (hours), from first dose of antimicrobial to LC-MS/MS result availability (hours), duration of the pre-analytical, analytical, and post-analytical phase (hours), therapeutic target attainment, 28-day all-cause mortality, ICU and hospital length of stay, proportions of participants with positive microbiological isolates, their susceptibilities, and resistance patterns to study antimicrobials. The analytical phase was defined from the time the blood samples were logged into the laboratory to the completion of testing. As this was a feasibility study of turnaround times for TDM, drug concentrations were not disclosed to clinical teams. While beta-lactam TDM is part of routine clinical care in some settings, there is limited guidance on how results should be interpreted in light of fixed dosing recommendations for piperacillin/tazobactam and meropenem by the Medicines and Healthcare products Regulatory Agency (MHRA) and the European Medicines Agency (EMA).^13,14^ Implementation of routine TDM was beyond the scope of this work package.

### Study procedures

Following enrolment, baseline data, including demographics (age, sex, body mass index), need for surgery during admission, indication for antimicrobial, physiological support (sequential organ failure assessment score, mechanical ventilation, vasopressor support, need for CRRT) and routinely measured biomarkers (white cell count, C-reactive protein, procalcitonin), were recorded by a trained member of the research team and blood samples collected from an indwelling arterial or central venous catheter over the duration of one antimicrobial dosing interval (either six or 8 h; intermittent boluses were standard of care at the institution at the time of study conduct). Blood sampling timings were prespecified and protocolised (Supplemental Table 1). Both piperacillin/tazobactam and meropenem were administered as 30-min intermittent infusions at the recruiting sites during the study period; this was not modified for the purpose of this observational study. Blood samples were processed according to a study-specific sampling manual; this included immediate transfer of samples to the laboratory, centrifugation, aliquoting, and freezing to −80°C, with TDM blood samples taken directly to an on-site research laboratory for drug quantification using LC-MS/MS. As per the study sampling manual, all samples were frozen within an hour of collection, unless directly quantified with LC-MS/MS. Drug concentrations in serum samples were quantified using a previously published method.^15^ The quantification analysis was performed on a Waters Acquity Class I TQD system with a Waters BEH C18 column maintained at 60°C. Results were not released to the clinical team due to the observational nature of the study, an approach approved by the ethics committee. Follow-up data on outcomes and routinely collected microbiological culture data were collected at 28 days following enrolment. All study data were collected and managed using REDCap electronic data capture tools hosted at Manchester University NHS Foundation Trust.^16,17^

### Sample size

We did not undertake a formal sample size calculation as this was a feasibility study. We planned to enrol 30 participants with complete follow-up data, which enabled an assessment of recruitment feasibility for a future study with similar eligibility criteria and procedures, whilst providing sufficient data for PK modelling as part of our planned secondary analyses.

### Statistical analyses

We calculated central tendency and spread for continuous variables using mean and standard deviation (SD) for normally distributed data, and median and interquartile range (IQR) for non-normally distributed data. We evaluated data using visual inspection of histograms, and tested normality using the Shapiro-Wilk test and skewness statistics. We considered *p* values of <0.05 as statistically significant. A timeline of key events was plotted. All statistical analyses and plots were generated in R (version 4.2.3).

## Results

**Figure 1.**
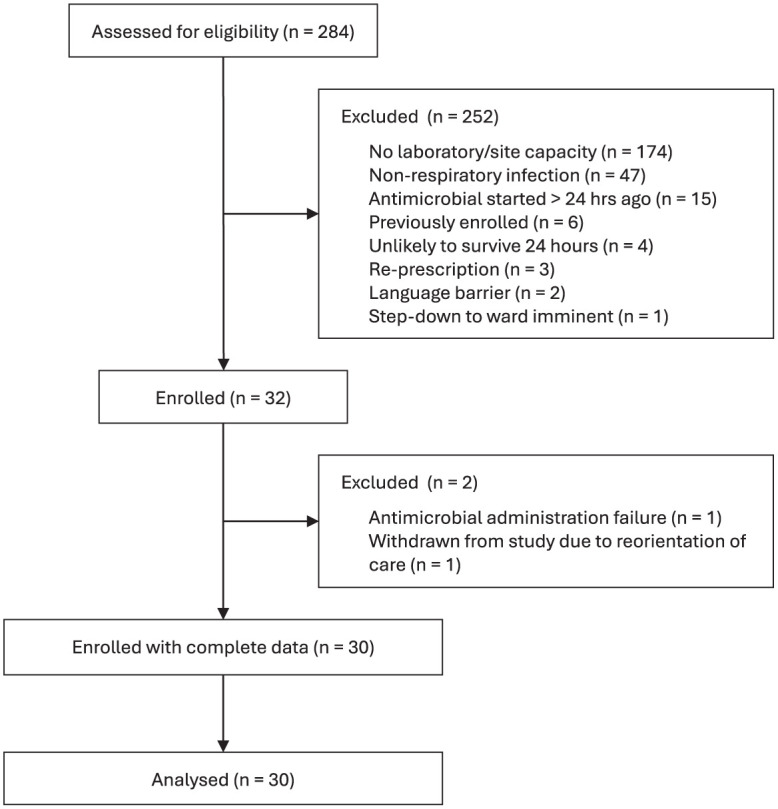
Study flow diagram.

We recruited 30 participants with full follow-up data. Two participants were excluded following enrolment: one deteriorated rapidly following inclusion and was excluded due to reorientation of care goals, and one was excluded due to a prolonged administration of antimicrobial dose. There was one protocol violation, where the subsequent dose was administered prior to fourth sample being taken. The study flow diagram is presented in Figure 1. Baseline characteristics of included participants are described in Table 1, with complete reasons for ineligibility available in Supplemental Table 2.

**Table 1.** Participant baseline characteristics according to prescribed antimicrobial.

Groups	Piperacillin/tazobactam	Meropenem	All
	(*n* = 26)	(*n* = 4)	(*n* = 30)
Age (median, IQR)	63 (55–76)	62 (56–69)	63 (55–75)
Sex (number, %)			
Male	14 (54%)	2 (50%)	16 (53%)
Female	12 (46%)	2 (50%)	14 (47%)
Body mass index (median, IQR)	29.8 (24.0–35.8)	30.1 (27.7–33.4)	29.8 (24.7–35.8)
Surgery during admission (number, %)			
Yes	17 (65%)	1 (25%)	18 (60%)
No	9 (35%)	3 (75%)	12 (40%)
Antimicrobial indication (number, %)			
HAP	20 (77%)	2 (50%)	22 (73%)
Sepsis^a^	2 (7.7%)	0 (0%)	2 (6.7%)
VAP	1 (3.8%)	1 (25%)	2 (6.7%)
CAP	1 (3.8%)	0 (0%)	1 (3.3%)
Other^b^	2 (7.7%)	1 (25%)	3 (10%)
SOFA score (median, IQR)	6 (3–8)	9 (8.5–10)	6 (3–9)
Mechanical ventilation (number, %)	15 (58%)	4 (100%)	19 (63%)
Vasopressor support (number, %)	7 (27%)	2 (50%)	9 (30%)
CRRT (number, %)	1 (4%)	2 (50%)	3 (10%)
Biomarkers at baseline (median, IQR)			
White cell count × 10^9^/L	12.6 (9.98–15.8)	21.4 (19.5–22.3)	13.6 (10.3–16.2)
C-reactive protein, mg/L	218 (141–281)	143 (63–221)	211 (133–272)
Procalcitonin, ng/mL^c^	0.51 (0.28–1.25)	15.2^c^	0.63 (0.29–2.55)

CAP: community-acquired pneumonia; CRRT: continuous renal replacement therapy; HAP: hospital-acquired pneumonia; IQR: interquartile range; SOFA: sequential organ failure assessment; VAP: ventilator-associated pneumonia.

a Likely respiratory origin.

b Diagnoses included: aspiration pneumonia (1), exacerbation of bronchiectasis (1), neutropaenic sepsis (1).

c Baseline procalcitonin was measured in 15 participants in the piperacillin/tazobactam group, and one participant in the meropenem group.

### Primary outcome

TDM results from LC-MS/MS were available within two dose intervals for 20 (67%) participants (Table 2). Twelve (40%) participants were successfully enrolled prior to the first dose of antimicrobial being administered.

**Table 2.** Primary and secondary outcomes.

Outcomes	Piperacillin/tazobactam (*n* = 26)	Meropenem (*n* = 4)	All (*n* = 30)
Primary outcome			
TDM result available within two dosing intervals from sampling (number, %)	17/26 (65%)	3/4 (75%)	20/30 (67%)
Secondary outcomes			
Time from sampling to TDM result (median, IQR)^a^	10.6 (9.6–20.2)	21.4 (17.9–22.5)	10.9 (9.6–21.4)
Time from antimicrobial course start to TDM result (median, IQR)^a^	25.6 (10.6–30.9)	24.6 (22.0–29.8)	25.4 (12.3–30.9)
Duration of pre-analytical phase in hours (median, IQR)^a^	8.1 (7.7–17.9)	18.9 (15.3–20.2)	8.3 (7.7–18.8)
Duration of analytical phase in hours (median, IQR)	2.3 (1.9–2.7)	2.4 (2.2–2.8)	2.3 (2.0–2.7)
Survival at 28 days (number, %)	20 (77%)	1 (25%)	21/30 (70%)
ICU length of stay, days (median, IQR)	6.5 (5–23)	-^b^	7 (5–25)
Hospital length of stay, days (median, IQR)	17 (10–27)	-^b^	17 (10–27)

ICU: intensive care unit; IQR, interquartile range; TDM: therapeutic drug monitoring.

a Median (IQR) values calculated for minutes and converted to hours.

b All patients in the meropenem group were still hospitalised at the time of follow-up. One patient was discharged from ICU 23 days following admission.

### Secondary outcomes

**Figure 2.**
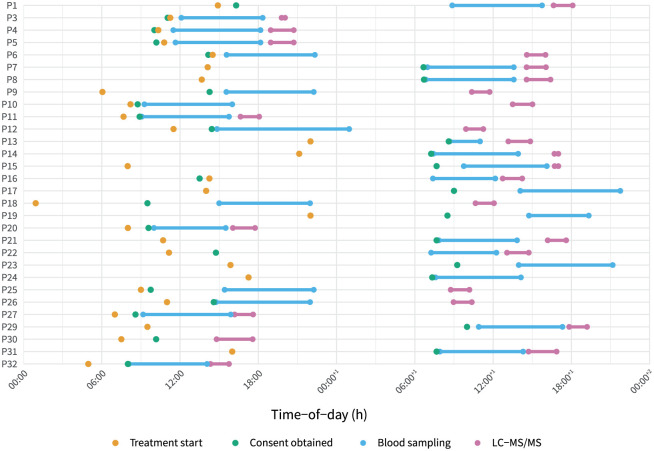
Timing of key events for enrolled participants. Each row represents an individual participant (P1–P32). Time-of-day is shown on the x-axis, spanning 2 calendar days. Key time points are marked as follows: treatment start (orange dot), consent obtained (green dot), blood sampling period (blue lines with dots at start and end), and sample processing (pink lines with dots at start and end). This visualisation demonstrates heterogeneity in timing of sample collection relative to treatment and consent, highlighting logistical variability in sample processing across participants and time of day. P17, P19, P23 and P24 had drug quantification performed on the third calendar day of treatment, at 47.2, 135.1, 42.8, and 70.6 h, respectively.

The median (IQR) time from blood sampling to TDM result and from antimicrobial course start to TDM result was 10.9 (9.6–21.4) h and 25.4 (12.3–30.9) h, respectively. The pre-analytical and analytical phases spanned a median (IQR) of 8.3 (7.7–18.8) h and 2.3 (2.0–2.7) h, respectively. Times of key study events are presented at participant level in Figure 2.

At 28 days, 21 (70%) participants were alive, 15 were discharged from ICU and 9 were discharged from hospital, with a median (IQR) 7 (5–25) day and 17 (10–27) day ICU and hospital length of stay, respectively. Primary and secondary outcomes are summarised in Table 2.

Twenty-one participants (70%) had positive microbiological isolates from the time of their treatment episode. The most commonly isolated bacterial species were *Escherichia* spp. (*n* = 5) and *Staphylococci* (*n* = 5). *Candida* spp. were isolated in four participants. A detailed list of isolated pathogens is reported in Supplemental Table 3. Susceptibilities were available for 13/21 (62%) of culture-positive participants, with pathogens resistant to the respective study antimicrobials identified in 4/21 (19%).

Screening and enrolment data, including temporal patterns of antimicrobial prescribing during the study period, are reported in Supplemental Figures 4–8.

## Discussion

In this prospective feasibility study, we found that real-time beta-lactam TDM within two dosing intervals is operationally achievable in critically ill patients with lower respiratory infections. Furthermore, we were able to recruit the prespecified number of study participants to a complex time-sensitive intervention at a consistent rate. Most importantly, the findings of our study offer useful insights to support the design of a larger future interventional trial of beta-lactam TDM in critical care. While this study focussed on operational feasibility, timely TDM could ultimately support improved antimicrobial stewardship and positively impact patient outcomes in critical care.

Our findings should be considered in the context of existing data from RCTs evaluating beta-lactam TDM in critically ill patients.^8–10,18,19^ Whereas some studies managed to deliver actionable TDM results within 6 h during weekdays,^18^ most did not explicitly target early sampling and result delivery. The DOLPHIN study, a single-centre trial of model-informed precision dosing to guide treatment, took first samples at varying timepoints, with a limit at 36 h, followed by a 12-h turnaround target for the quantification assay.^10^ Whereas the TARGET trial, a multicentre RCT of beta-lactam TDM in 249 septic patients, achieved a median recruitment time of 15 h, the time to result availability or target attainment was not explicitly reported.^8^ Similarly to our study, they excluded participants where TDM was not achievable within 24 h of randomisation. Studies have cumulatively not demonstrated a clear positive impact on robust patient outcomes, with delays to intervention implementation as one potential reason for this.^20^ Arguably, the most unwell critically ill patients who may be more likely to benefit from optimal antimicrobial exposures may not survive beyond the 48-h mark. A recently published prospective observational study of 268 critically ill patients treated with beta-lactams supported by TDM noted an association with earlier TDM and improved cure rates, with timing of TDM noted as a significant predictor of clinical cure and increases in dose to achieve higher exposures predicting lower 30-day mortality.^21^

It is important to acknowledge that several centres outside the UK already employ beta-lactam TDM as a matter of routine, with professional society recommendations supporting this practice.^22,23^ Whereas routine TDM may be useful for detecting overexposure, prompting a dose decrease, or providing reassurance when concentrations are within range, there is a paucity of clear guidance on how dosing adjustments should be made in the face of subtherapeutic beta-lactam levels when patients are already receiving the maximum licenced dose. This gap in practical dosing strategies highlights a critical barrier to optimising TDM implementation at scale. The real-world effectiveness and feasibility of daily beta-lactam TDM is the subject of a current ongoing study with a published protocol.^24^ Presently, routine clinical beta-lactam TDM is not a round-the-clock service in the NHS, and considerable infrastructure investment would be required for this to become the case, likely harnessing a hub-and-spoke model to optimise resource use.

Patients who may benefit from TDM present at all times of day. Although our study was designed around a ‘working hours’ provision of antimicrobial quantification in the research setting, with participant enrolment reflective of said availability, we still encountered considerable delays to results. It is important to distinguish delays stemming from *research* of TDM as an intervention and the operational delays were TDM implemented as *standard of care*. Both reflect limited availability of skilled personnel outside of regular working hours; however, the latter could be ameliorated through technological advances as described below. Importantly, clinical trials of beta-lactam TDM in critically ill patients would need to account for both sources of delays to reduce the likelihood of missing a real effect difference (type II error).

Our study has several limitations. As a single-centre observational study with a relatively small sample size, findings may not be generalisable to other settings, especially those with robust existing clinical TDM pathways. Eligibility criteria included presumed or confirmed lower respiratory infection, which may have introduced an element of bias; we did not use post hoc independent adjudication to assess whether an infection was likely to be present or not as this was not within the scope of the study. The limited availability of out-of-hours LC-MS/MS for research at our laboratory may have introduced bias, as most participants were excluded due to lack of laboratory capacity. Whereas participants could have been included at any time of day with samples frozen and processed at the next available timeslot, such a delay would have rendered results devoid of practical utility. Considering clinical implementation, our data on timings should be interpreted cautiously, as delays associated with consent or rigid per-protocol sampling requirements may have skewed the timings; prospective observational studies following implementation would help address this knowledge gap. Although we only observed an enrolment rate of 11%, this was likely due to the very high ascertainment of potential participants as a result of using automated research notifications. Such participant discovery rates reflect continuous screening by the electronic health record, and are therefore more reflective of the actual population that stands to benefit from inclusion in research. Furthermore, with rapid real-time ascertainment of potentially eligible individuals, we were able to recruit 40% of our cohort prior to or at the point of receipt of the first dose of antimicrobial (Figure 2).

Future research should prioritise larger, multicentre trials assessing whether real-time TDM coupled with rapid dose adjustment can improve clinical outcomes such as mortality, organ dysfunction, and resource use. Key operational barriers, including sample transport, laboratory processing times, and result reporting, should be addressed, potentially through near-patient TDM solutions, integrated automated laboratory infrastructure, and improved electronic decision support tools. Deferred consent research frameworks, with waivers of consent at individual level, are required to ensure timely participant recruitment into trials. Patient selection strategies should focus on subgroups most likely to benefit, such as those with augmented renal clearance, septic shock, or at risk of multidrug-resistant infections requiring higher exposures, based on previously identified covariates.^25^ Additionally, trials should incorporate economic analyses to determine the cost-effectiveness of routine TDM implementation.^26^ The findings of our study support the feasibility of enrolling participants into a time-sensitive trial prior to or at the point of receipt of the first dose of antimicrobial. Our group is currently setting up both observational and interventional studies of beta-lactam TDM and model-informed precision dosing.

## Conclusion

We demonstrated feasibility of recruiting critically ill patients to a time-sensitive clinical study involving real-time complex monitoring. Timely feedback of beta-lactam TDM results to clinicians is achievable, however, logistical constraints, such as the availability of specialist staff and equipment outside of regular working hours, may continue to impede widespread implementation. Future clinical trials of beta-lactam TDM in critical care should take turnaround times into consideration at the design stage.

## Supplemental Material

sj-docx-1-inc-10.1177_17511437251404324 – Supplemental material for A prospective cohort feasibility study of real-time beta-lactam antimicrobial therapeutic drug monitoring in critically ill patients with lower respiratory infection: The TDM-TIME study Supplemental material, sj-docx-1-inc-10.1177_17511437251404324 for A prospective cohort feasibility study of real-time beta-lactam antimicrobial therapeutic drug monitoring in critically ill patients with lower respiratory infection: The TDM-TIME study by Jan Hansel, Jake Lain, Emmanuel O. Erhieyovwe, Aybaniz Ismayilli, James Orr, Brian G. Keevil, Kayode Ogungbenro, Paul M. Dark and Timothy W. Felton in Journal of the Intensive Care Society
