# Supplementary material for: A prospective cohort feasibility study of real-time beta-lactam antimicrobial therapeutic drug monitoring in critically ill patients with lower respiratory infection: The TDM-TIME study
Source: J Intensive Care Soc. 2025 Dec 24;27(1):22–9. doi: 10.1177/17511437251404324 (PMC12738274; doi:10.1177/17511437251404324)
Supplement: sj-docx-1-inc-10.1177_17511437251404324 – Supplemental material for A prospective cohort feasibility study of real-time beta-lactam antimicrobial therapeutic drug monitoring in critically ill patients with lower respiratory infection: The TDM-TIME study [file sj-docx-1-inc-10.1177_17511437251404324.docx]

**Supplemental materials**

**A prospective cohort feasibility study of real-time beta-lactam antimicrobial therapeutic drug monitoring in critically ill patients with lower respiratory infection: The TDM-TIME Study**

Jan Hansel^1^, Jake Lain^2^, Emmanuel Erhieyovwe^3^, Aybaniz Ismayilli^4^, James Orr^1^, Brian G. Keevil^2^, Kayode Ogungbenro^5^, Paul M. Dark^1^, Timothy W. Felton^1^

1 Division of Immunology, Immunity to Infection and Respiratory Medicine, The University of Manchester, Manchester, UK

2 NIHR Centre for Precision Approaches to Combatting Antimicrobial Resistance, Manchester University NHS Foundation Trust, Manchester, UK

3 Acute Intensive Care Unit, Manchester University NHS Foundation Trust, Manchester, UK

5 Division of Pharmacy & Optometry, The University of Manchester, Manchester, UK

Corresponding author: Dr Jan Hansel

jan.hansel@manchester.ac.uk

[Table S1. Blood sample collection timing table for piperacillin/tazobactam and meropenem 3](#_Toc196745968)

[Table S2. Summary of reasons for ineligibility 4](#_Toc196745969)

[Table S3. Isolated pathogens from period of treatment episode with counts and sources 5](#_Toc196745970)

[Figure S4. Box-plot of time to screening completion according to enrolment status 6](#_Toc196745971)

[Figure S5. Heatmap of prescription frequencies according to hour of the day and day of the week 7](#_Toc196745972)

[Figure S6. Piperacillin/tazobactam and meropenem prescriptions according to time of day and day of week 8](#_Toc196745973)

[Figure S8. Piperacillin/tazobactam and meropenem prescriptions according to time of day 9](#_Toc196745974)

Supplemental Table 1. Blood sample collection timing table for piperacillin/tazobactam and meropenem

| **Meropenem TDS (q8hrs)** | | |  | **Piperacillin/tazobactam QDS (q6hrs)** | | |
| --- | --- | --- | --- | --- | --- | --- |
|  | | |  |  | | |
| *Sample name* | *Volume* | *Time from dose* |  | *Sample name* | *Volume* | *Time from dose* |
| ID_TDM-1 | 2 x 1.2 ml | **01:00** |  | ID_TDM-1 | 2 x 1.2 ml | **01:00** |
| ID_TDM-2 | 2 x 1.2 ml | **03:00** |  | ID_TDM-2 | 2 x 1.2 ml | **02:30** |
| ID_TDM-3 | 2 x 1.2 ml | **05:00** |  | ID_TDM-3 | 2 x 1.2 ml | **04:00** |
| ID_TDM-4 | 2 x 1.2 ml | **08:00** |  | ID_TDM-4 | 2 x 1.2 ml | **06:00** |
|  | | | | | | |
| **Meropenem BD (q12hrs)** | | |  | **Piperacillin/tazobactam TDS (q8hrs)** | | |
|  | | |  |  | | |
| *Sample name* | *Volume* | *Time from dose* |  | *Sample name* | *Volume* | *Time from dose* |
| ID_TDM-1 | 2 x 1.2 ml | **01:00** |  | ID_TDM-1 | 2 x 1.2 ml | **01:00** |
| ID_TDM-2 | 2 x 1.2 ml | **03:00** |  | ID_TDM-2 | 2 x 1.2 ml | **03:00** |
| ID_TDM-3 | 2 x 1.2 ml | **06:00** |  | ID_TDM-3 | 2 x 1.2 ml | **05:00** |
| ID_TDM-4 | 2 x 1.2 ml | **12:00** |  | ID_TDM-4 | 2 x 1.2 ml | **08:00** |

Supplemental Table 2. Summary of reasons for ineligibility

| Reason for ineligibility | Count (%)  (n = 254) |
| --- | --- |
| No capacity | 174 (69) |
| Non-respiratory infection | 47 (19) |
| Antimicrobial started > 24 hrs ago | 15 (5.9) |
| Previously enrolled | 6 (2.4) |
| Unlikely to survive 24 hours | 4 (1.6) |
| Re-prescription | 3 (1.2) |
| Non-English speaker | 2 (0.8) |
| Screening failure | 1 (0.4) |
| Stepdown to ward imminent | 1 (0.4) |
| Withdrawn from study | 1 (0.4) |

Supplemental Table 3. Isolated pathogens from period of treatment episode with counts and sources

| Pathogen | N | Source |
| --- | --- | --- |
| **Bacteria** | | |
| *Acinetobacter* sp.^a^ | 1 | Sputum |
| *Corynebacterium* | 1 | Sputum |
| *Enterobacter* sp.^a^ | 1 | Sputum |
| *Escherichia coli*^a^ | 5 | Blood (1), Bronchial washing/BAL (4) |
| *Haemophilus influenzae* | 2 | Bronchial washing/BAL (2) |
| *Klebsiella* spp. | 2 | Bronchial washing/BAL (2) |
| *Pseudomonas aeruginosa* | 1 | Sputum |
| *Serratia marcescens* | 2 | Blood (1), Bronchial washing/BAL (1) |
| *Staphylococcus aureus* | 2 | Bronchial washing/BAL (1), Sputum (1) |
| *Staphylococcus capitis* | 1 | Blood |
| *Staphylococcus epidermidis* | 2 | Blood (1), Bronchial washing/BAL (1) |
| *Stenotrophomonas* sp. | 1 | Bronchial washing/BAL |
| *Streptococcus pneumoniae* | 1 | Sputum |
| **Viruses^b^** | | |
| *Adenovirus* | 1 | Sputum |
| *Human metapneumovirus* | 1 | Sputum |
| *Parainfluenza-3* | 1 | Sputum |
| *Rhinovirus* | 1 | Sputum |
| **Fungi** | | |
| *Aspergillus* spp. | 2 | Sputum (2) |
| *Candida* spp. | 4 | Bronchial washing/BAL (2), Sputum (2) |
| Other yeast | 1 | Sputum |

BAL: bronchoalveolar lavage

^a^ Resistant strains isolated

^b^ Isolated using polymerase chain reaction (PCR) testing

Supplemental Figure 4. Box-plot of time to screening completion according to enrolment status

Supplemental Figure 5. Heatmap of prescription frequencies according to hour of the day and day of the week


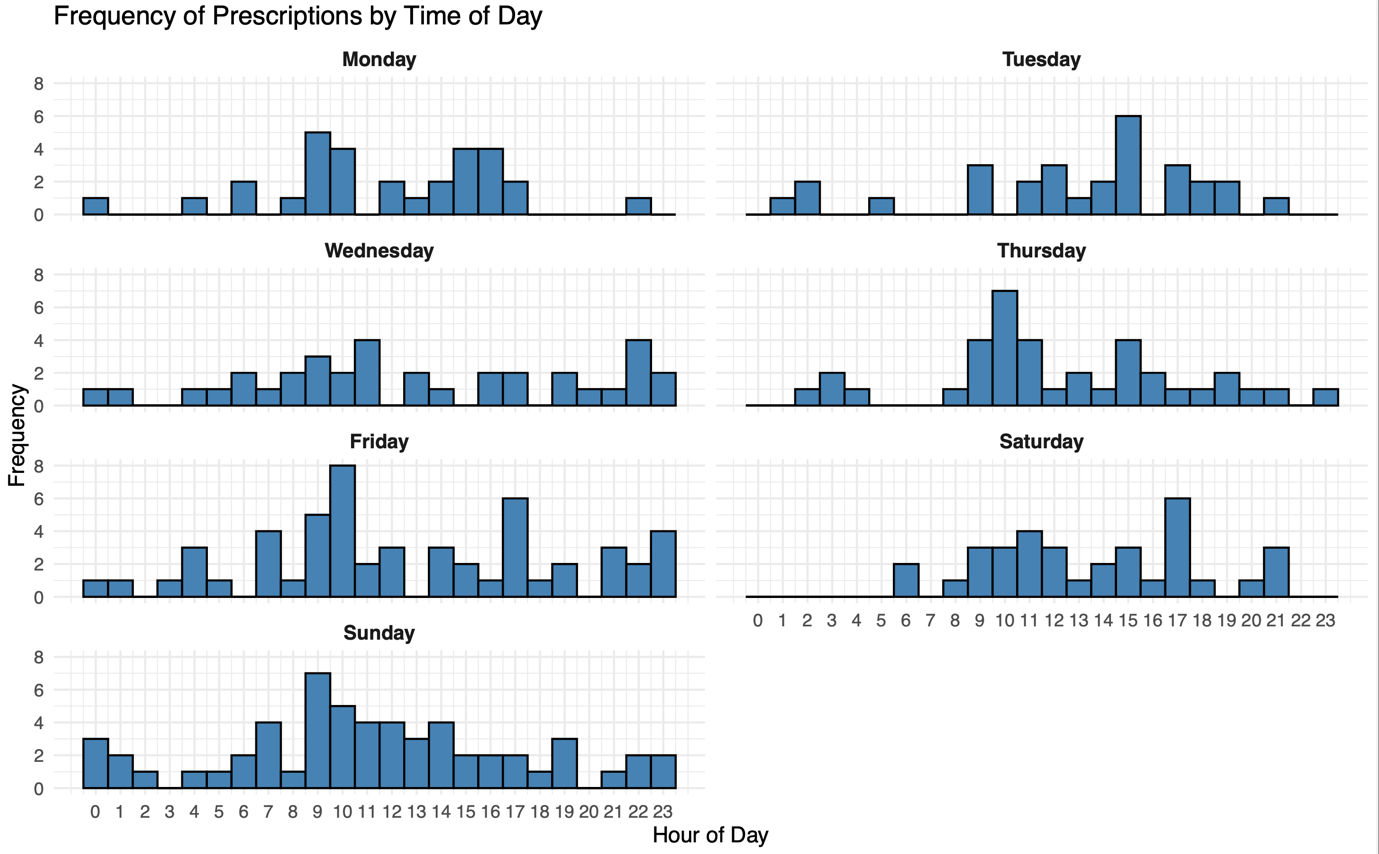


Supplemental Figure 6. Piperacillin/tazobactam and meropenem prescriptions according to time of day and day of week

Supplemental Figure 8. Piperacillin/tazobactam and meropenem prescriptions according to time of day

There are two notable spikes, consistent with the periods of morning clinical reviews (9:00-10:00), followed by the ward round (15:00-17:00), which also includes an infectious diseases/microbiology liaison review (n = 276).

**STROBE Statement—checklist of items that should be included in reports of observational studies**

|  | Item No. | Recommendation | Page  No. | Relevant text from manuscript |
| --- | --- | --- | --- | --- |
| **Title and abstract** | 1 | (*a*) Indicate the study’s design with a commonly used term in the title or the abstract | p. 1 | “prospective cohort feasibility study” |
|  |  | (*b*) Provide in the abstract an informative and balanced summary of what was done and what was found | p. 2 | See abstract |
| Introduction | | | |  |
| Background/rationale | 2 | Explain the scientific background and rationale for the investigation being reported | p. 3 | See introduction |
| Objectives | 3 | State specific objectives, including any prespecified hypotheses | pp. 3-4 | “This study aimed to investigate turnaround times for delivering actionable results from TDM of beta-lactam antimicrobials in patients admitted to the intensive care unit with severe lower respiratory infections. Furthermore, we sought to identify technology-enabled approaches to improve participant recruitment into future time-sensitive TDM trials.” |
| Methods | | | |  |
| Study design | 4 | Present key elements of study design early in the paper | p. 4 | “single-centre prospective observational cohort feasibility study” etc |
| Setting | 5 | Describe the setting, locations, and relevant dates, including periods of recruitment, exposure, follow-up, and data collection | p. 4 | “conducted at two ICUs in Manchester, UK, between 12 December 2023 and 21 June 2024” |
| Participants | 6 | (*a*) *Cohort study*—Give the eligibility criteria, and the sources and methods of selection of participants. Describe methods of follow-up  *Case-control study*—Give the eligibility criteria, and the sources and methods of case ascertainment and control selection. Give the rationale for the choice of cases and controls  *Cross-sectional study*—Give the eligibility criteria, and the sources and methods of selection of participants | pp. 4-5 | Inclusion and exclusion criteria listed, follow-up at 28 days |
|  |  | (*b*) *Cohort study*—For matched studies, give matching criteria and number of exposed and unexposed  *Case-control study*—For matched studies, give matching criteria and the number of controls per case | NA | NA |
| Variables | 7 | Clearly define all outcomes, exposures, predictors, potential confounders, and effect modifiers. Give diagnostic criteria, if applicable | pp. 4-5 | Outcomes defined |
| Data sources/ measurement | 8* | For each variable of interest, give sources of data and details of methods of assessment (measurement). Describe comparability of assessment methods if there is more than one group | pp. 5-6 | Definitions of procedures and outcomes |
| Bias | 9 | Describe any efforts to address potential sources of bias | p. 6 | Information on release of clinical results |
| Study size | 10 | Explain how the study size was arrived at | p. 6 | Sample size rationale |

| Quantitative variables | 11 | Explain how quantitative variables were handled in the analyses. If applicable, describe which groupings were chosen and why | p. 6 | Statistical analysis described; no groupings of variables |
| --- | --- | --- | --- | --- |
| Statistical methods | 12 | (*a*) Describe all statistical methods, including those used to control for confounding | p. 6 | Statistical analysis described |
|  |  | (*b*) Describe any methods used to examine subgroups and interactions | NA | NA |
|  |  | (*c*) Explain how missing data were addressed | NA | NA |
|  |  | (*d*) *Cohort study*—If applicable, explain how loss to follow-up was addressed  *Case-control study*—If applicable, explain how matching of cases and controls was addressed  *Cross-sectional study*—If applicable, describe analytical methods taking account of sampling strategy | NA | NA |
|  |  | (*e*) Describe any sensitivity analyses | NA | None reported, no inferential statistics |
| Results | | | | |
| Participants | 13* | (a) Report numbers of individuals at each stage of study—eg numbers potentially eligible, examined for eligibility, confirmed eligible, included in the study, completing follow-up, and analysed | p. 7, fig. 1 | “30 participants with full follow-up data. Two participants were excluded following enrolment: one deteriorated rapidly following inclusion and was excluded due to reorientation of care goals, and one was excluded due to a prolonged administration of antimicrobial dose.” |
|  |  | (b) Give reasons for non-participation at each stage | p. 7, fig. 1 | As above |
|  |  | (c) Consider use of a flow diagram | fig. 1 | Flow diagram |
| Descriptive data | 14* | (a) Give characteristics of study participants (eg demographic, clinical, social) and information on exposures and potential confounders | table 1 | See table of baseline characteristics |
|  |  | (b) Indicate number of participants with missing data for each variable of interest | table 1 | Missingness reported for procalcitonin values |
|  |  | (c) *Cohort study*—Summarise follow-up time (eg, average and total amount) | p. 7, table 2 | 28 days |
| Outcome data | 15* | *Cohort study*—Report numbers of outcome events or summary measures over time | p. 7, table 2 | Reported for primary and all secondary outcomes |
|  |  | *Case-control study—*Report numbers in each exposure category, or summary measures of exposure | NA | NA |
|  |  | *Cross-sectional study—*Report numbers of outcome events or summary measures | NA | NA |
| Main results | 16 | (*a*) Give unadjusted estimates and, if applicable, confounder-adjusted estimates and their precision (eg, 95% confidence interval). Make clear which confounders were adjusted for and why they were included | NA | No inference |
|  |  | (*b*) Report category boundaries when continuous variables were categorized | NA | No categories |
|  |  | (*c*) If relevant, consider translating estimates of relative risk into absolute risk for a meaningful time period | NA | No relative risks reported |

| Other analyses | 17 | Report other analyses done—eg analyses of subgroups and interactions, and sensitivity analyses | NA | No sensitivity analyses undertaken |
| --- | --- | --- | --- | --- |
| Discussion | | | | |
| Key results | 18 | Summarise key results with reference to study objectives | p. 8 | “In this prospective feasibility study, we found that real-time beta-lactam TDM within two dosing intervals is operationally achievable in critically ill patients with lower respiratory infections. Furthermore, we were able to recruit the prespecified number of study participants to a complex time-sensitive intervention at a consistent rate. Most importantly, the findings of our study offer useful insights to support the design of a larger future interventional trial of beta-lactam TDM in critical care. While this study focused on operational feasibility, timely TDM could ultimately support improved antimicrobial stewardship and positively impact patient outcomes in critical care.” |
| Limitations | 19 | Discuss limitations of the study, taking into account sources of potential bias or imprecision. Discuss both direction and magnitude of any potential bias | p. 10 | “Our study has several limitations. As a single-centre observational study with a relatively small sample size, findings may not be generalisable to other settings, especially those with robust existing clinical TDM pathways. Eligibility criteria included presumed or confirmed lower respiratory infection, which may have introduced an element of bias; we did not use post hoc independent adjudication to assess whether an infection was likely to be present or not as this was not within the scope of the study. Finally, the limited availability of out-of-hours LC-MS/MS for research at our laboratory may have introduced bias, as most participants were excluded due to lack of laboratory capacity. Whereas participants could have been included at any time of day with samples frozen and processed at the next available timeslot, such a delay would have rendered results devoid of practical utility. Conversely, there are some strengths to our study. Although we only observed an enrolment rate of 11%, this was likely due to the very high ascertainment of potential participants as a result of using automated research notifications. Furthermore, with rapid real-time ascertainment of potentially eligible individuals, we were able to recruit 40% of our cohort prior to or at the point of receipt of the first dose of antimicrobial (Figure 2).” |
| Interpretation | 20 | Give a cautious overall interpretation of results considering objectives, limitations, multiplicity of analyses, results from similar studies, and other relevant evidence | p. 11 | “We demonstrated feasibility of recruiting critically ill patients to a time-sensitive clinical study involving a real-time complex diagnostic.” |
| Generalisability | 21 | Discuss the generalisability (external validity) of the study results | p. 11 | “Timely feedback of beta-lactam TDM results to clinicians is achievable, however, logistical constraints, such as the availability of specialist staff and equipment outside of regular working hours, may continue to impede widespread implementation.” |
| Other information | |  | | |
| Funding | 22 | Give the source of funding and the role of the funders for the present study and, if applicable, for the original study on which the present article is based | p. 12 | “The TDM-TIME Study was supported by an Intensive Care Society New Investigator Award to JH and the National Institute for Health and Care Research (NIHR) Manchester Biomedical Research Centre (BRC) (NIHR203308). JH is funded by the NIHR through a Doctoral Fellowship (NIHR304654) and was previously supported by an NIHR Academic Clinical Fellowship. The views expressed in this publication are those of the author(s) and not necessarily those of the NIHR, NHS or the UK Department of Health and Social Care. JL is funded by the NIHR Manchester BRC (NIHR203308).” |

*Give information separately for cases and controls in case-control studies and, if applicable, for exposed and unexposed groups in cohort and cross-sectional studies.

**Note:** An Explanation and Elaboration article discusses each checklist item and gives methodological background and published examples of transparent reporting. The STROBE checklist is best used in conjunction with this article (freely available on the Web sites of PLoS Medicine at http://www.plosmedicine.org/, Annals of Internal Medicine at http://www.annals.org/, and Epidemiology at http://www.epidem.com/). Information on the STROBE Initiative is available at www.strobe-statement.org.
